# How does disgust regulate social rejection? a mini-review

**DOI:** 10.3389/fpsyg.2023.1141100

**Published:** 2023-06-16

**Authors:** John A. Terrizzi, Richard S. Pond, Trevor C. J. Shannon, Zachary K. Koopman, Jessica C. Reich

**Affiliations:** ^1^Department of Psychology, Texas Woman’s University, Denton, TX, United States; ^2^Department of Psychology, University of North Carolina Wilmington, Wilmington, NC, United States

**Keywords:** disgust, shame, behavioral immune system, social rejection, social exclusion, ostracism

## Abstract

The need to belong is a fundamental aspect of human nature. Over the past two decades, researchers have uncovered many harmful effects of social rejection. However, less work has examined the emotional antecedents to rejection. The purpose of the present article was to explore how disgust––an emotion linked to avoidance and social withdrawal––serves as an important antecedent to social rejection. We argue that disgust affects social rejection through three routes. First, disgust encourages stigmatization, especially of those who exhibit cues of infectious disease. Second, disgust and disease-avoidance give rise to cultural variants (e.g., socially conservative values and assortative sociality), which mitigate social interaction. Third, when the self is perceived as a source of contamination, it promotes shame, which, subsequently, encourages withdrawal from social interaction. Directions for future research are also discussed.

## Introduction

Humans are intensely social. People enjoy immense benefits, both psychologically and physically, from their participation in and maintenance of positive relationships (Baumeister and Leary, 1995). Consequently, social rejection serves as a serious threat to our mental and physical well-being. Although much is known about the consequences of rejection, including poorer self-control, increased self-defeating and aggressive behavior, and even physiological responses associated with pain (e.g., Holt-Lunstad et al., 2010; DeWall et al., 2011; Eisenberger, 2011; Leary, 2015), less is well-known about its antecedents. In the present article, we review empirical evidence that suggests disgust––an emotion linked to avoidance and social withdrawal––is an important precursor to rejection. We argue that disgust is directly and indirectly linked to social rejection through a few key pathways.

We obtained articles for this review by searching the PsychINFO database and Google Scholar for published articles, using the terms: “disgust OR parasite stress OR disease threat OR behavioral immune system” AND “rejection OR exclusion OR ostracism OR avoidance.” These broad terms allowed us to cast a wide net to identify relevant literature regarding the influence of disgust on rejection.

## The adaptive value of social connection

Long before dating apps and Facebook, our early ancestors faced a world much harsher than our own, in which pursuing a solitary existence posed grave danger (Buss, 2008). To offset the risks of solitude, early humans formed small communities wherein members helped each other to survive (e.g., hunting, foraging, building shelter, defense from physical threats, childcare duties; Trivers, 1971; Eastwick, 2009). Given how critical group-living was for our ancestors, they required psychological mechanisms for detecting and responding to social threats (Leary and Downs, 1995; Kurzban and Leary, 2001; Leary, 2001; Chester et al., 2012; Wesselmann et al., 2012). For instance, social pain likely evolved concurrently with early societies to promote survival (MacDonald and Leary, 2005). Studies examining neural responses to socially painful events have largely supported a model of common neural substrates for detecting physical and social pain (see Eisenberger, 2011 for a comprehensive review). Psychological mechanisms for detecting social threats are even sensitive to non-verbal cues (e.g., averted eye gaze) that may warn of potential rejection (Wirth et al., 2010).

Rejection as a social strategy likely developed alongside early group living and has been molded by selective pressures to satisfy certain adaptive needs (Williams, 1966; Cosmides and Tooby, 1994; Kurzban and Leary, 2001). Of course, our brains would likely require certain mechanisms through which to address the specific issues emerging from sociality (Wesselmann et al., 2012). Disgust is potentially one mechanism for strategically triggering social rejection to avoid costly group members (or trigger social withdrawal when the costly group member is oneself).

## Disgust as an antecedent to rejection

Here, we discuss three routes by which disgust affects social rejection. First, disgust encourages stigmatization (see Oaten et al., 2011). It encourages avoidance and rejection of others (Park et al., 2003; Faulkner et al., 2004; Navarrete and Fessler, 2006; Park et al., 2006; Terrizzi et al., 2010), especially those who exhibit cues of infectious disease (Van Leeuwen and Petersen, 2018). Second, disgust and disease-avoidance (i.e., parasite stress) give rise to cultural variants (e.g., socially conservative values and assortative sociality; see Fincher and Thornhill, 2012; Terrizzi et al., 2013; Thornhill and Fincher, 2014), which mitigate social interaction. Third, disgust can promote self-isolation. When the self is perceived as a source of contamination (i.e., self-disgust; see Overton et al., 2008), it promotes shame (Terrizzi and Shook, 2020), which encourages withdrawal from social interaction (Tangney et al., 1996). We preface our discussion of these routes by outlining the adaptive challenge of infectious disease and its evolutionary solutions (i.e., the physiological immune system and the behavioral immune system).

## The adaptive challenge of infectious disease

Infectious diseases present an adaptive challenge for humans. Like all living organisms, infectious agents (e.g., viruses, bacteria) are in the business of survival and reproduction. However, their reproductive success can make us sick or even die. Consequently, humans and pathogens are locked in an evolutionary arms race (see Nesse and Williams, 1994). Pathogens are evolving new methods of decoding our security system, and we are evolving new tactics for fending them off. One of our lines of defense is the physiological immune system (PIS). When an infectious agent enters the human body, the PIS produces antibodies that recognize specific portions of the pathogens, bind to them, and, hopefully, inactivate them (Delves and Roitt, 2000). Although the PIS is an effective tool for combating pathogens, it can be costly, sometimes resulting in friendly fire (i.e., attacking the very body that it is designed to protect). Fortunately, the PIS is not the only solution to the problem of infectious disease. We are also equipped with psychological mechanisms that help promote disease-avoidance.

## The behavioral immune system

The behavioral immune system (BIS) is the first line of defense against infectious disease (Schaller, 2006). It is a suite of psychological mechanisms that promote disease-avoidance. The goal of the BIS is to limit exposure to infectious agents. It helps us avoid situations, people, and objects that would increase the likelihood of infection exposure. The BIS accomplishes this by triggering adaptive cognitive (e.g., infection specific thoughts), affective (e.g., disgust), and behavioral responses (e.g., avoidance, repulsion), which collaborate to produce prophylactic responses to cues of infectious disease (Schaller and Duncan, 2007).

## The embodied cognitive nature of disgust

Disgust is a key component of the BIS. It is a cross-cultural human emotion (Ekman, 1970) that is believed to have originated in our ancestral past as a means of distinguishing healthy and edible items from those that may be dangerous (Rozin and Fallon, 1987). As such, the facial expression accompanying disgust results in flaring nostrils, squinted eyes, protruding tongue, and a gaping mouth, which aid in minimizing disease exposure (Rozin et al., 1994). Though there is cross-cultural variability in the triggers of disgust, some triggers like bodily by-products (e.g., blood, feces, vomit) seem to be cross-culturally universal (Curtis and Biran, 2001; Curtis et al., 2011). This is not all surprising, given how such substances often spread diseases. Indeed, pictures that are disease-relevant (e.g., resembling bodily fluids) are more cross-culturally evocative of disgust than those that are not disease-relevant (e.g., blue slime; Curtis et al., 2004).

From an evolutionary perspective, psychological systems that are designed to solve adaptive challenges are not always accurate. Rather, they promote the avoidance of errors that are the most reproductively costly (see Haselton and Buss, 2000). In the case of the BIS, this means that individuals will be more vulnerable to Type I errors (i.e., perceiving an object as a disease threat when it is not; Oaten et al., 2009). As a result, people are prone to “magical contagion” (Rozin et al., 1992). For example, participants will avoid eating fudge that is shaped like dog feces (Rozin et al., 1986).

Conceptually, disgust is a system that is turned on and off by environmental triggers (i.e., cues of infectious disease). When individuals are exposed to a disgusting object (e.g., rotten meat), it elicits disgust, which encourages disease-avoidant behavior. The salience of bodily by-products as a universal disgust elicitor is indicative of the role that human-to-human contact plays in the transmission of infectious disease. Contagious diseases are often spread by incidental contact with bodily by-products (e.g., respiratory droplets). Given the prevalence of this route of infectious exposure, it follows that disgust will have implications for human social interaction. In the next few sections, we will discuss how disgust and disease-avoidance cause stigmatization and avoidance of others.

## Disgust and stigmatization

Disgust is an avoidant emotion (Cottrell and Neuberg, 2005). Consequently, its influence on social behavior should be indicative of social conservatism, rejection, and avoidance of others (especially those who are different). Evidence of the avoidant nature of disgust and its impact on social behavior can be seen in the aggression literature. Disgust is negatively associated with physical aggression (i.e., approach-oriented aggression; Pond et al., 2012) but positively associated with relational aggression (e.g., rejection; Molho et al., 2017).

One of the ways disgust encourages avoidance of others is through the mechanism of stigma. Stigmatization is the process of categorizing groups or individuals based on undesirable characteristics, both physical (e.g., morphological differences) and moral (e.g., norm violations), as a means of segregation and avoidance (see Goffman, 1963). From a disease-avoidance perspective, stigma can be conceptualized as a strategy for decreasing the probability of exposure to infectious disease by limiting contact with “contaminated” groups (see Oaten et al., 2011). As disgust makes us prone to Type I errors, the effect that disgust has on stigmatization will default toward false positives. Thus, groups or individuals that pose no disease-threat will be avoided.

The disease-avoidant nature of stigma impacts perceptions of disease-threat. For instance, stigmatized others (e.g., out-group members) are often blamed for the onset of epidemics (Oaten et al., 2011). Stigma also has vicious long-lasting downstream effects on social identity. Stigma is often placed on individuals or groups because of strong feelings of disgust and avoidance (Major and O’brien, 2005; Oaten et al., 2011). Once a particular group has been stigmatized, the mere label (i.e., social categorization) of that group can confer contamination concerns. Thus, the label itself can metaphorically contaminate those to whom it is applied.

Interestingly, although stigma leads to social rejection by out-group members, stigmas can also drive a stronger sense of association with in-group members (Major and O’brien, 2005; Oaten et al., 2011). When our sense of self is threatened and we are rejected by others, we seek to repair that by finding support from other members of our own, stigmatized group and relying more on the group identity to depersonalize the offense (Crocker et al., 1991).

## Disgust and in-group/out-group bias

Because other people are a significant source of contamination, humans attend to morphological differences that could signal disease-threat (disease cues: runny nose, swelling; Duncan et al., 2009). Some evidence suggests that attention to such cues can even trigger immunological responses that help prepare the body for disease (Schaller et al., 2010). Individuals who are particularly concerned with infectious disease show an over-perception of disease threat (i.e., perceiving and recalling disease cues where there were none; Miller and Maner, 2012).

As disgust is believed to be a disease-avoidance mechanism, it follows that it would trigger avoidance and rejection of those who exhibit cues of infectious disease (Van Leeuwen and Petersen, 2018). However, its effect on social interaction is not limited to those who display cues of infectious disease. Disgust seems to cause an in-group/out-group bias, such that it encourages avoidance of out-group members and, reciprocally, a greater affinity for in-group members. Disgust and disease-avoidant concerns are associated with prejudice and avoidance of a wide variety of out-group members, including foreigners, sexual minorities, and obese individuals (Park et al., 2003; Faulkner et al., 2004; Navarrete and Fessler, 2006; Terrizzi et al., 2010). In addition to its impact on interpersonal prejudice and avoidance, disgust seems to have a large impact on cultural values.

## Cultural quarantining

Culture plays an important role in the defense against infectious disease. Parasite stress theory suggests that historic exposure to infectious disease affects the evolution of cultural value systems (see Thornhill and Fincher, 2014). In areas of the world in which there are higher rates of infectious disease and more life lost due to infectious disease, there should be more orderliness and strict adherence to social norms. Indeed, regions with higher rates of infectious disease exhibit more constraints on high-risk behaviors (e.g., sexual behaviors, drug use; Fincher et al., 2008; Schaller and Murray, 2008), tend to be more collectivistic (i.e., a cultural orientation that encourages in-group cohesion and adherence to social norms; Guernier et al., 2004; Fincher et al., 2008), and experience more religiosity and assortative sociality (i.e., preference for similar others; Fincher and Thornhill, 2012).

The potential prophylactic value of tight cultures was also observed during the COVID-19 pandemic. Tight cultures (i.e., those that are more orderly and have less crime) exhibited lower mortality rates and less prevalence of COVID-19 (Gelfand et al., 2021). Likewise, power distance (i.e., the extent to which subordinates accept the power of authority figures) and institutional collectivism (which are both associated with norm adherence) were predictive of lower rates of COVID-19 morbidity and mortality (Kumar, 2021).

Not only are regional differences in parasite stress correlated with conservative cultural values (e.g., collectivism, adherence to social norms), but so too are individual differences in disgust sensitivity and concern about infectious disease (Terrizzi et al., 2013). Those who are more sensitive to disgust and chronically concerned about disease-threat are more likely to report higher levels of socially conservative values (e.g., right-wing authoritarianism, xenophobia, religious fundamentalism). Additionally, these value systems seem to function as a means of discouraging interaction with out-groups by promoting in-group assortative sociality (Terrizzi et al., 2010, 2012, 2014).

## Shame as self-directed disgust

Other people are not the only object of our disgust. Humans are self-conscious beings and, just as we make evaluations of others, we make evaluations of ourselves (i.e., self-esteem). Therefore, we can experience self-disgust, which has severe socioemotional consequences (e.g., depression and anxiety; Overton et al., 2008). Here we demonstrate how internalized disgust (i.e., self-disgust) can lead to self-stigmatization and self-rejection.

One of the consequences of self-reflected disgust is shame. Shame is a negatively valenced self-conscious emotion that results in global self-condemnation (Tangney, 1991; Niedenthal et al., 1994). In the case of self-disgust, global condemnation is perceiving the self as a source of contamination.

Though little research has explored the relation between disgust and shame, there is some preliminary evidence for their association. Evidence suggests that perceiving facial expressions of disgust can trigger increased shame (Giner-Sorolla and Espinosa, 2011). Specifically, across two cultures (i.e., the United Kingdom and Spain), participants primed with pictures depicting facial expressions of disgust reported more shame than guilt, and participants who saw angry faces reported more guilt than shame.

Not only does perceiving facial expressions of disgust induce shame, but those more sensitive to disgust and have a greater fear of contamination are more vulnerable to shame. In a series of studies, disgust sensitivity and fear of contamination were associated with shame but not guilt, and priming individuals with disgust increased shame but not guilt in individuals who were sensitive to disgust (Terrizzi and Shook, 2020).

Just as disgust stymies social interaction as a means of limiting exposure to infectious disease, so too may shame. Shame and disgust have similar behavioral features. They both encourage avoidance and social withdrawal. Just as disgust and disease-threat encourage behavioral avoidance (Faulkner et al., 2004; Navarrete and Fessler, 2006), shame that results from moral transgressions encourages avoidance of social interaction (Orth et al., 2006; Schmader and Lickel, 2006). Likewise, both shame and disgust seem to be involved in the maintenance of social norms. They are both described as moral emotions, which encourage moral behavior and adherence to social norms (Haidt, 2003; Tangney et al., 2007), and they are both associated with moral decision-making (Tangney et al., 2007; Schnall et al., 2008). Furthermore, deficiencies in both shame and disgust are associated with psychopathy (i.e., an antisocial disregard for social norms; Morrison and Gilbert, 2001; Tangney et al., 2003; Tybur et al., 2009).

Because disgust and shame both encourage social withdrawal (Faulkner et al., 2004; Navarrete and Fessler, 2006; Orth et al., 2006; Schmader and Lickel, 2006), it is likely that intense feelings of both emotions will precede and coincide with feeling lonely, rejected, and socially disconnected. That is, disgust should promote feelings of shame, which, in turn, increase perceptions of rejection.

## Discussion

Humans are tremendously social. Yet, the ironic consequence of this sociality is that social rejection is ubiquitous. It occurs everyday, and no one is immune to its harmful influence. To neatly encapsulate all the reasons for which rejection occurs is an ambitious endeavor. Humans evolved to avoid poor social exchange partners, favor their in-group (and exclude or exploit out-group members), and avoid contact with those who may be differentially likely to carry communicable pathogens. In each case, the tendency to exclude others confers survival advantages. The present review supports the idea that disgust plays an important role in the social rejection experience. Not only does this emotion trigger the avoidance of costly group members, but, when directed inward, it can result in shame, self-condemnation, and social withdrawal.

Although there is strong theoretical and empirical evidence that suggests that both disgust and shame play a critical role in human social rejection, there is room for further research. For instance, one limitation is that there is a dearth of experimental work demonstrating the extent to which disgust induces feelings of shame, and less still that identifies shame as a precursor to self-rejection and avoidance of others. Future work would benefit from manipulating disgust (both generally as well as self-disgust) and shame in the laboratory and then measuring their impact across multiple measures of rejection (both toward others and oneself).

Furthermore, we conceptualized disgust and shame as important antecedents of rejection; however, the bidirectionality of the relations is unclear. Some theoretical and empirical work has identified shame as a potential consequence of rejection (Leary, 2015; Wang et al., 2020). However, it appears that rejection does not modulate the disgust experience (Antico et al., 2018). Moreover, no work that we are aware of has identified disgust as a consequence of rejection, but rather a trigger (Park et al., 2003; Faulkner et al., 2004; Navarrete and Fessler, 2006; Terrizzi et al., 2010). Yet, the social rejection experience is complicated. The rejection literature is replete with experimental studies that focus on between-person differences within a single laboratory session. Consequently, only a few studies have examined how the experience of rejection develops within individuals over time (e.g., Nezlek et al., 2012). This is another significant limitation of the extant literature. Future work would benefit from exploring the day-to-day emotional experiences that unfold and coincide with perceived rejection and related phenomena (e.g., feelings of loneliness and disconnection, discrimination, ostracism).

Finally, the literature is dominated by Western samples of college students that are predominately White and female. Thus, it is difficult to know the extent to which results can be generalized to other populations. Future work would benefit from obtaining more diverse community samples, as well as greater cross-cultural representation.

## Author contributions

All authors listed have made a substantial, direct, and intellectual contribution to the work and approved it for publication.

## Conflict of interest

The authors declare that the research was conducted in the absence of any commercial or financial relationships that could be construed as a potential conflict of interest.

## Publisher’s note

All claims expressed in this article are solely those of the authors and do not necessarily represent those of their affiliated organizations, or those of the publisher, the editors and the reviewers. Any product that may be evaluated in this article, or claim that may be made by its manufacturer, is not guaranteed or endorsed by the publisher.
